# Hemobilia caused by pancreatic arteriovenous malformation: A case report and literature review

**DOI:** 10.1097/MD.0000000000013285

**Published:** 2018-12-14

**Authors:** Xiaolei Liu, Jia Huang, Haidong Tan, Zhiying Yang

**Affiliations:** Department of General Surgery, China–Japan Friendship Hospital, Beijing 100029, China.

**Keywords:** arteriovenous malformation, gastrointestinal bleeding, hemobilia

## Abstract

**Rationale:**

Hemobilia caused by arteriovenous malformation is extremely rare but could be lethal. To date, most reports have been single-case reports, and no literature reviews are available.

**Patient concerns:**

A 47-year-old man presented to the emergency department with abdominal pain and fever. He complained of abdominal pain and weight loss for the past 2 months.

**Diagnoses:**

Contrast-enhanced computed tomography and magnetic resonance imaging showed a heterogenous lesion located in pancreatic head and tumor was suspected.

**Interventions:**

Endoscopic retrograde cholangiopancreatography was performed and bleeding from papilla of Vater could be viewed. Nasobiliary drainage was placed to alleviate the pain and jaundice. Emergency laparotomy was performed due to the recurrence of severe pain and bleeding, and pancreatoduodenectomy was then performed. Macroscopic examination showed the ulceration connected with collected vessels which were located in pancreatic head and microscopic examination confirmed the presence of arteriovenous malformation.

**Outcomes:**

The patient recovered uneventfully and was discharged 10 days after the surgery. He is asymptomatic on 4-month follow up.

**Lessons:**

Arteriovenous malformation is a rare cause of hemobilia, but it could lead to life threatening bleeding. Transarterial embolization could be effective to control the bleeding temporarily, however repeated hemorrhage may occur. Surgical resection may be a better option.

## 1 Introduction

Hemobilia is defined as bleeding into the biliary system and is an uncommon source of upper gastrointestinal (GI) bleeding. Approximately two-third of hemobilia cases result from medical interventions.^[1]^ Percutaneous liver biopsy and transhepatic cholangiography are the most common causes of injury to the liver vasculature resulting in hemobilia. In addition, blunt or penetrating trauma to the liver can also cause hemobilia. Arteriovenous malformation (AVM) of pancreas or common bile duct (CBD) is a vascular anomaly in which blood flows from the arterial system directly into the portal venous system without passing through the capillaries. Though it may be asymptomatic, some cases might present with a spectrum of symptoms including abdominal pain, signs of acute pancreatitis and even catastrophic episodes of GI bleeding.^[2]^ Hemobilia caused by AVM is extremely rare but could be lethal. Instead of transarterial embolization (TAE) which is commonly used to treat hemobilia, surgical resection is the most common treatment modality for patients with GI bleeding caused AVM.^[3–5]^ Herein, we report a case of massive hembilia caused by AVM.

## 2 Case report

**Figure 1:**
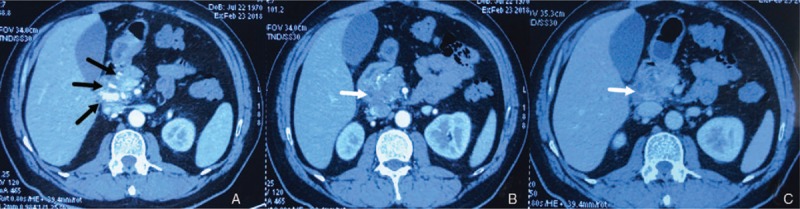
Contrast-enhanced CT Scan. Multiple, high-density, dot-like enhancing vascular structures could be detected during arterial phase ([A] marked with black arrows). A heterogenous lesion was also showed in pancreatic head both in arterial and venous phases ([B and C] marked with white arrows). CT = computed tomography.

**Figure 2:**
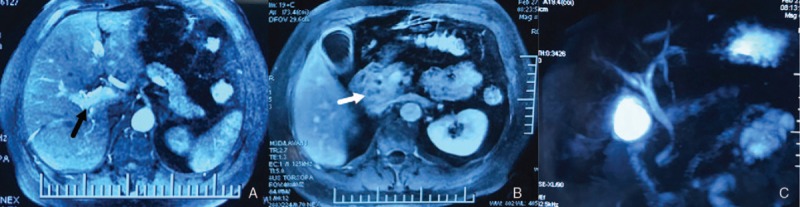
Contrast-enhanced MRI and MRCP. Early portal venous filling during the arterial phase ([A] marked with black arrow) and a heterogenous lesion located in pancreatic head were detected ([B] marked with white arrow). No dilation or filling defect of common bile duct was found (C). MRCP = magnetic resonance cholangiopancreatography, MRI = magnetic resonance imaging.

**Figure 3:**
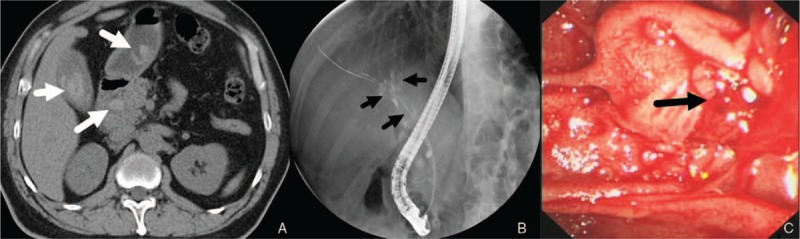
CT scan and ERCP. High-density masses with the suspicion of blood clots were showed in gallbladder, stomach and common bile duct ([A] marked with black arrows). Multiple filling defects in the common bile duct were showed on ERCP ([B] marked with white arrows). Bleeding from papilla of Vater could be directly seen during ERCP ([C], marked with black arrow). CT = computed tomography, ERCP = endoscopic retrograde cholangiopancreatography.

**Figure 4:**
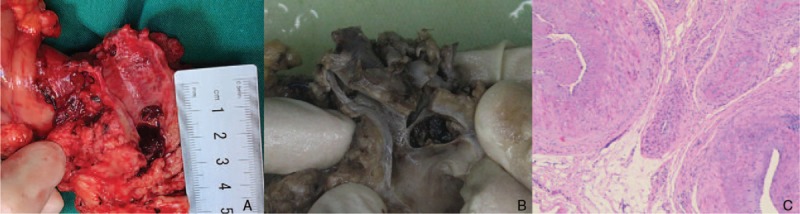
Surgical specimen and histopathology. A huge ulceration was seen in the distal common bile duct (A), which connected with the honey-comb like collected vessels which were located in pancreatic head (B). Microscopic examination confirmed the presence of arteriovenous malformation (C).

A 47-year-old man presented to the emergency department with abdominal pain and fever. He complained of abdominal pain and weight loss for the past 2 months. His medical history was negative. On physical examination, the abdomen was tender to palpation in the right upper quadrant. Laboratory data showed elevated level of white blood cell (11.23×10^9^/L), alanine aminotransferase (427 IU/L), aspartate aminotransferase (410 IU/L), total bilirubin (TB, 34.57 μmol/L), direct bilirubin (DB, 25.01 μmol/L), glutamyl transpeptidase (1159 IU/L), alkaline phosphatase (416 IU/L) and carbohydrate antigen-199 (84.68 IU/ml). The level of hemoglobin (Hb, 147 g/L), other biochemistry, blood tumor markers, and coagulation studies were all within the normal range. Contrast-enhanced computed tomography (CT) of the abdomen showed a heterogenous lesion of pancreatic head and multiple, high-density, dot-like enhancing vascular structures (Fig. 1). Contrast-enhanced magnetic resonance imaging (MRI) of the abdomen and cholangiopancreatography (MRCP) showed early portal venous filling during the arterial phase, a heterogenous lesion located in pancreatic head, but no dilation or filling defect of CBD (Fig. 2). Since pancreatic tumor was suspected, lesion biopsy with endoscopic ultrasonography was attempted. However, the procedure failed due to the intolerance of the patient and the abdominal pain got worse. Another CT scan was performed and high-density masses were detected in gallbladder and CDB, which were not showed on previous CT scan (Fig. 3A). Then, hemobilia was suspected and repeated blood test showed mild anemia (Hb 117 g/L) and further elevation of bilirubin (TB 110.02 μmol/L, DB 56.68 μmol/L).The patient continued to have severe abdominal pain which was speculated to be due to obstruction of bile duct. Hence endoscopic retrograde cholangiopancreatography (ERCP) was performed. The result showed multiple filling defects in the CBD, which were not showed on precious MRCP (Fig. 3B). Bleeding from papilla of Vater could be directly seen during ERCP (Fig. 3C). Nasobiliary tube was placed to alleviate the pain and jaundice. After the procedure, pain was alleviated and the serum bilirubin was decreased. Both AVM and pancreatic tumor were considered as the probable diagnosis, and surgical resection was planned. However, two days later after ERCP, the abdominal pain got severe again and the level of Hb fell to 61 g/L. Laparotomy was performed immediately. There were no abnormal findings of the liver, gallbladder, stomach, spleen, or bowel. The head of pancreas was soft but enlarged. Pancreatoduodenectomy was then performed and the unfixed specimen showed a huge ulceration of distal CBD (Fig. 4A). Macroscopic examination showed the ulceration connected with the honey-comb like collected vessels which were located in pancreatic head and microscopic examination confirmed the presence of arteriovenous malformation (Fig. 4B and C). The patient recovered uneventfully and was discharged 10 days after the surgery. He is asymptomatic on 4-month follow up.

Informed consent was obtained from the patient regarding the publication of his clinical details and images. The study was approved by the Institutional Ethical Review Board of China–Japan Friendship Hospital.

## 3 Literature search and retrieval

We searched the PubMed and EMBASE databases to retrieve relevant articles published from 1987 to January 2018. The keywords used included “arteriovenous malformation,” “hemobilia,” and “gastrointestinal bleeding.” A total of 7 articles on hemobilia caused by AVM have been published over the past 30 years.^[3–9]^ A total of 7 cases were reported, all men, with a mean age of 54.6 years (47–66 years). All of the patients had abdominal pain, but only 3 patients had classic symptomatic triad of hemobilia (abdominal pain, jaundice and GI bleeding). AVM were located in pancreas in 3 patients (2 patients in whole pancreas and 1 patient in pancreatic head), in common bile duct in 2 patients. The location of AVM in the other 2 patients was described in pancreatobiliary region. Four patients received drainage of bile duct, including 3 cases of endoscopic nasobiliary drainage (ENBD) and 1 case of percutaneous transhepatic biliary drainage (PTCD). TAE was performed for 4 patients to control the bleeding, while 2 of them received surgical resection finally. A total of 3 patients received surgical treatment, including 2 cases of pylorus-preserving pancreatoduodenectomy and 1 case of total pancreatoduodenectomy and splenectomy. One patient received ablation with alcohol injection by angiography and the other patient received symptomatic treatment. All the patients were treated successfully and discharged uneventfully, but only 3 reports had the results of follow-up (Table 1).

**Table 1:**
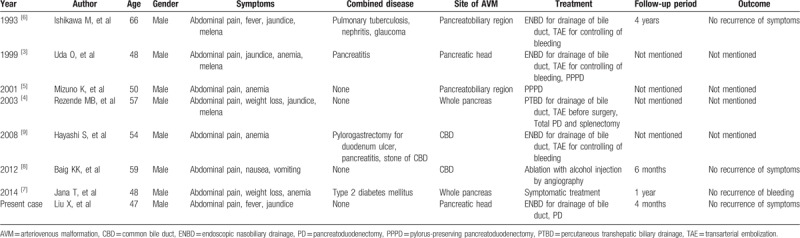
Case reports of hemobilia caused by AVM.

## 4 Discussion

Hemobilia is defined as bleeding into the biliary system and is an uncommon source of upper GI bleeding and the first case of hemobilia was reported in 1654 by Glisson.^[1]^ The pathophysiology of hemobilia occurs when there is a fistula between a blood vessel and the biliary tree. The classic presentation triad of hemobilia includes right upper quadrant pain, GI bleeding, and jaundice, but only a small part of patients presented with the classic triad.^[10–12]^ It is mostly characterized by minor hemorrhage that could stop spontaneously. In some cases, massive hemorrhage may occur, presenting as melena or hematemesis, and bleeding through the ampulla of Vater could be directly viewed through endoscopic examination,^[13]^ like the case we reported. In the cases with minor hemobilia, blood clots have a tendency to form in the bile duct and cause biliary obstruction, which may result in abdominal pain, elevation of liver enzymes, and the presentation of jaundice. In the past, reports of hemobilia cases showed that accidental trauma, either penetrating or blunt, was the most common etiology.^[14–16]^ However, trauma as the predominant etiology has changed, and iatrogenic causes involving bile duct procedures are now reported as the most common cause. These include procedures such as of percutaneous transhepatic cholangiography, liver biopsy, PTBD, ERCP, or transjugular intrahepatic portosystemic shunt.^[17–21]^ This shift is mainly due to the increased use of these procedures. Iatrogenic injury accounts for over 50% of hemobilia cases in most series now.^[22]^ Other causes of hemobilia include gallbladder and bile duct stones, biliary varices, biliary parasites, benign, and malignant tumors involving the biliary tree, liver surgery, pancreatitis, and hepatitis.^[23–25]^

AVM refers to a complex tangle of abnormal arteries and veins linked by one or more direct connections called fistulas or shunt.^[26]^ AVM may be divided into two types: (1) Congenital AVM, which arise from an anomalous differentiation in the rudimentary plexus of primordial blood vessels. These lesions tend to be multiple. (2) Acquired AVM, which are usually secondary to inflammation, tumor, or trauma.^[27,28]^ AVM can occur anywhere in the body. An increasing number of patients with AVM in the digestive organs are being diagnosed due to widespread use of imaging techniques. Many cases of pancreatic AVM are associated with Rendu-Osler-Weber syndrome and are known to be a part of the visceral angiodysplasia of hereditary hemorrhagic telangiectasia.^[9]^ The most frequently involved portion of the pancreas has been reported to be the head (59.4%), followed by the body and tail (33.3%) and the entire pancreas (7.2%).^[2]^ The majority of patients with pancreatic AVM remain asymptomatic, but some present with abdominal pain or GI bleeding. GI bleeding has been reported to be fatal in 30 to 50% of patients with pancreatic AVM.^[29,30]^ Five possible mechanisms have been proposed as underlying GI bleeding in patients with pancreatic AVM: bleeding from eroded vessels in the gastrointestinal mucosa caused by close contact and compression by the AVM, bleeding from duodenal ulcer caused by local ischemic injury of the duodenal mucosa by local infarction resulting in abnormal vessels in the pancreatic AVM which is close contact with the duodenum, bleeding from gastroesophageal varices caused by portal hypertension resulting from arterialization of portal veins, hemosuccus pancreaticus caused by the rupture of abnormal vessels of the pancreatic AVM into the pancreatic duct, hemobilia caused by the rupture of abnormal vessels of the pancreatic AVM into the bile duct.^[31,32]^ The angiography is useful for the diagnosis of the AVM and subsequent interventional therapy can be performed after the diagnosis. But the technique is invasive. Recent advances in noninvasive radiologic methods such as CT, MRI, and endoscopic ultrasonography have made diagnosis of AVM safer and accurate.^[28,30,32]^ The contrast-enhanced CT or MRI findings which suggested AVM features included strong enhancement or conglomeration of small hypervascular spots in the lesion, and early contrast filling of the portal vein (thus during the arterial phase), which could be also viewed in this case we reported.

However, AVM is a very rare cause of hemobilia. To the best of our knowledge, only 8 cases with hemobilia caused by AVM have been reported in the English language literature and the site of AVM includes pancreas and CBD.^[3–9]^ The reported treatment for AVM involved symptomatic treatment, TAE, irradiation and surgical resection. TAE has been shown to be of clinical utility in the management of congenital AVM. Although it has been suggested that permanent embolic agents should be used and an effort made to extinguish the hemangiomatous portion of the AVM, the embolization of multiple vessels is quite difficult, and the risk of enteric necrosis is great. Generally, pancreatic AVM has multiple feeding arteries, making it very difficult to achieve complete embolization.^[33,34]^ Recurrent bleeding has been reported in patients treated by embolization alone.^[6,35]^ Moreover, the natural history of this disease, due to the propensity for growth of new collateral vessels, may lead to repeated hemorrhage and the progressive development of portal hypertension. Catastrophic episodes of gastrointestinal bleeding occurred in 50% of the reported patients with pancreatic AVMs.^[29,30,36]^ The longest follow-up period for hemobilia patients treated by TAE was only 4 years^[3]^ and the result of long-term follow-up is still lacked. Surgical resection of the affected pancreas is the most effective treatment of patients with symptomatic pancreatic AVM. Song et al^[2]^ reported 12 symptomatic patients with pancreatic AVM, and 11 (91.7%) underwent pancreatic resection. During a median follow-up time of 37 months, no patient experienced recurrence and any major postoperative complication. For the 8 hemobilia patients caused by AVM, 4 (50%) of them received surgical treatment finally. However, there is no report on long-term result.

In summary, AVM is a rare cause of hemobilia, but could lead to life threatening bleeding. TAE could be effective to control the bleeding temporarily, but repeated hemorrhage may occur. For these patients, surgical resection may be a better option.

## Author contributions

**Conceptualization:** Xiaolei Liu, Zhiying Yang.

**Data curation:** Haidong Tan.

**Investigation:** Jia Huang, Haidong Tan.

**Visualization:** Jia Huang.

**Writing – original draft:** Xiaolei Liu.

**Writing – review & editing:** Zhiying Yang.
